# Effects of temporary vascular occluder poloxamer 407 Gel on the endothelium

**DOI:** 10.1186/1749-8090-8-16

**Published:** 2013-01-22

**Authors:** Arif Gucu, Ilkin Cavusoglu, Cuneyt Eris, Faruk Toktas, Tugrul Goncu, Ahmet Ozyazicioglu

**Affiliations:** 1 Department of Cardiovascular Surgery, Bursa Yuksek Ihtisas Training and Research Hospital, Bursa, Turkey; 2 Department of Histology and Embryology, Uludag University Faculty of Medicine, Bursa, Turkey

**Keywords:** Endothelial damage, Poloxamer 407, Scanning electron microscopy, OPCAB

## Abstract

**Background:**

Coronary occlusion techniques during OPCAB may lead to an endothelial damage to the target vessel. The adverse effects of these techniques are well-known, and researches have been trying to find out new materials to occlude the coronary artery without an endothelial damage. In the present study, we investigate to the endothelial damage in the rat aorta which is occluded by Poloxamer 407 gel.

**Methods:**

Forty-five rats were randomized in three groups: (1) segment of the aorta was occluded with Poloxamer 407 gel in P 407 group; (2) segment of the aorta was occluded with microvascular clamp in MV clamp group; and (3) no onclusion was available in the Control group. The rats were sacrificed of observation, and a 15mm segment of the aorta was obtained as a specimen. Integrity of the endothelial lining was observed with a scanning electron microscopy.

**Results:**

Scanning electron microscopy revealed a statistically significant difference among the 3 groups (p<0,001) using the SPSS 13.0 test. No difference was found between the Control group and the P 407 group (p=0,059). The differences between MV clamp–Control group (p<0,001) and MV clamp–P 407 group were statistically significant (p<0,002).

**Conclusions:**

We suggest that Poloxamer 407 gel occlusion may be a safer and more effective method compared to the microvascular clamp occlusion.

## Background

Coronary artery occlusion techniques during OPCAB often result in suboptimal visualization due to bleeding from arteriotomy site. This leads to an increase in surgical trauma and risk the quality of the anastomosis. Recently, techniques and devices have been employed to improve the visibility during OPCAB surgery, including elastic snare sutures, microvascular clamps, intracoronary shunts and high flow gas insufflation materials. However, these techniques and devices all have a potential for mechanical damage to the coronary endothelium [1-7].

Despite improvements in hemostatic devices used in OPCAB surgery, the perfect device which allows a complete control of bleeding without generating coronary endothelial injury is yet to be introduced for use. On the other hand, patients undergoing coronary surgery already have atherosclerotic coronary arteries; therefore, bear endothelial damage to some extent [8]. Using the aforementioned methods for optimal visualization during OPCAB may even increase this endothelial damage. Thus, it is crucial to use the safest occlusion technique during OPCAB.

Poloxamers are a broad group of surfactants widely used in various industrial applications. These water-soluble, nontoxic and inert surfactants are tribloc copolymer of polyethylene oxide_a_–polypropylene oxide_b_–polyethylene oxide_a_. Different hydrophilic/hydrophobic ratios and physical characteristics can be obtained by varying the block size and total molecular weight of the poloxamers [9]. In particular, Poloxamer 407 - a polymer with a ratio of 70% polyoxyethylene and 30% polyoxypropylene - shows inverse thermo sensitivity [10]. Therefore, aqueous polymer solutions - with greater than 12% critical concentration - are liquid at low temperatures. However, it is notable that they turn into gel form at higher temperatures. The polymer is highly soluble; therefore, gel plug erodes in blood, and the dissolution time depends on how much polymer is injected, the concentration of the polymer in the solution, and the surrounding temperature [11]. This nontoxic polymer is not absorbed or metabolized and does not lead to an embolism. It is excreted urinary with a half-life of approximately 25 hours [12]. The use of Poloxamer 407 in vascular occlusion during OPCAB surgery has been previously reported [11,13-15]. In this study, we aimed to investigate the endothelial damage caused by Poloxamer 407 gel occlusion in the rat aorta using scanning electron microscopy.

## Methods

### Animals and surgical technique

Forty-five Wistar Albino female rats (3–4 months old, weighing 300–400 grams) were used in this experimental study. Following Uludag University Animal Experiments Local Ethic Committee approval (15.02.2011, ethics committee decision no: 2011-02-01), the study was conducted in accordance with the ‘Guide for the Care and Use of Laboratory Animals 2010’.

All of the rats were anesthetized with ketamine hydrochloride, 20 mg/kg IM (Ketalar® 500 mg, Pfizer, New York, USA) and xylazine hydrochloride, 15 mg/kg IM (Rompun® Inj. Solution 2%, Bayer, Leverkusen, Germany). In light of median laparotomy and exploration and dissection of the abdominal aorta the following procedures were performed:

1) P 407 group: after arteriotomy of the abdominal aorta, we placed a cardiac control syringe (Pluromed Inc, Lincoln, MA, USA) into the proximal part of the aorta. The distal part of the catheter (made of polypropylene and styrene butadiene rubber) is smooth with an olive-shaped ending taken as atraumatic for the endothelium. After the introduction of the catheter 15–20 mm into the proximal segment of rat abdominal aorta, 0.15 cc of Poloxamer 407 gel (LeGoo®, 22,5%, Pluromed Inc, Lincoln, MA, USA) was injected rapidly in order to avoid uneven coating of the gel, which could compromise the effectiveness of the occlusion. Lack of blood flow from the proximal direction in a few seconds meant that Poloxamer 407 gel became solid, and the plug occluded the vessel successfully. Ten minutes following complete occlusion, we irrigated the vessel gently with a cold saline solution to dissolve the plug in a few seconds. Subsequently, the vessel was carefully dissected free from the adherent tissue in a no-touch technique and was cut in transverse fashion 5 mm away from the occlusion area on each side. Hence, approximately 15-mm segment of the aorta was excised and taken as a specimen from the P 407 group.

2) MV clamp group: Following arteriotomy of the abdominal aorta, the proximal segment of arteriotomy was clamped with a microvascular clamp (Micro DeBakey bulldog clamp, Malden, MA, USA). After waiting for approximately 10 minutes, the vessel was harvested in the same fashion as in P 407 group and was taken as a specimen from the MV clamp group.

3) Control group: Following exploration, the vessel was harvested in the same fashion as in both groups, and was taken as a specimen from the Control group.

### Tissue procurement and preparation for SEM

The specimens of aorta were dissected longitudinally, pinned on cork plates, and fixed with 5% glutaraldehyde (in 0.13 M phosphate buffer, pH 7,2) at +4°C for 4 hours. Secondary fixation was performed with 1% OsO_4_ (in 0,13 M phosphate buffer, pH 7,2) at +4°C for 1 hour. The specimens were dehydrated in graded alcohol series, treated with 1/3, 1/1 and 3/1 alcohol/amylacetate mixtures and transferred to pure amylacetate. The tissue samples were kept in pure amylacetate for 2 days and then dried in critical point dryer (Baltec CPD Critical Point Dryer) and, coated with gold-palladium (Baltec SCD 005 Spotter Coater). The endothelial surfaces of the samples were investigated through a scanning electron microscope (Carl Zeiss EVO 40, Carl Zeiss SMT AG, Germany). The specimens were examined by a histologist who was experienced in scanning electron microscopy and, was blinded to all of the other data.

### Histological investigation

Histomorphologic changes of the endothelial layer were classified into four grades, and were described in Table 1.

**Table 1:** The classification of histomorphological changes of the endothelial layer

**Damage type**	**Histomorphological features**
**No damage**	The endothelial cells are contact with each other, neither change in the cellular morphology nor any decrease in size.
**Type 1 damage**	Cellular integrity of endothelial cells is unchanged. Endothelial cells are in contact with each other, but the flattening of the endothelial cells, changes in diameter and the occurrence of the nuclei. In addition, the adhesion of blood cells on the endothelium.
**Type 2 damage**	Detachment of the cells at their junction points and/or isolated absence of endothelial cells.
**Type 3 damage**	Appearance of the subendothelial tissue with desquamation of endothelial cells.

### Statistical analysis

Data were analyzed using SPSS 13.0 (SPSS, Inc, Chicago, USA). Three dependent groups were compared by Freedman Test, and the dual groups were compared by the Wilcoxon Test. Results which were p<0,05 were considered as significant. Descriptive values were noted as Median (Max-Min).

## Results

The specimens were examined with a scanning electron microscope and scored in accordance with the aforementioned classification criteria. Number of samples with different types of endothelial damage among control, Poloxamer 407 gel and microvascular clamp groups were described in Table 2.

**Table 2:** Number of samples with different types of endothelial damage in control, Poloxamer 407 gel and microvascular clamp groups

	**Control (n:15) group**	**MV Clamp (n:15) group**	**Poloxamer 407 Gel (n:15) group**
**No damage**	**15**	**-**	**10**
**Type I damage**	**-**	**10**	**4**
**Type II damage**	**-**	**4**	**1**
**Type III damage**	**-**	**1**	**-**

Freedman test revealed a statistically significant difference among the 3 groups (p<0,001). However, there was not any difference between the Control group and P 407 group (p=0,059) using the Wilcoxon test. The differences between MV clamp–Control group (p<0,001) and MV clamp–P 407 group were statistically significant (p<0,002).

## Discussion

One of the basic principles of the OPCAB is to establish a perfect anastomosis. On the other hand, one of the most important problems in OPCAB is the suboptimal visualization of the anastomosis site because of the bleeding through arteriotomy site despite the occlusion of the proximal coronary artery. The bleeding in the anastomosis site decreases the quality of the anastomosis and increases the probability of the surgical trauma [1]. For the improvement of the optimal visualization, the native coronary artery blood flow is usually occluded by the elastic snare suture or microvascular clamp. In spite of the occlusion of the proximal coronary artery, collateral flow impairs the visualization of the anastomosis site and distal portion of the coronary artery occlusion may be required. This is not recommended to avoid the possibility of the formation of the late term vascular stenosis and intimal damage [1]. Additional methods are employed to have an optimal visualization of the anastomosis site. These methods include intracoronary shunt or occluders and using high flow gas insufflation. Adverse effects of these methods are well reported [1-6]. In an experimental study, Gucu et al. [4] have found that filtrated high flow room air insufflation to the coronary anastomotic area may frequently cause type 1 and rarely type 2 endothelial damage. Intracoronary shunts may pose structurally and a functionally permanent endothelial damage [5] and high-flow gas insufflation may bear the risk of gas embolism and/or an endothelial damage [16].

The endothelium is a barrier between the vascular smooth muscle and blood that produces enzymes for activating and deactivating cardiovascular hormones and also the producer of relaxing and contracting factors. Vascular endothelium is located between the circulating the vascular smooth muscle and blood. Endothelial cells release various vasoactive substances regulating the function of vascular smooth muscle and trafficking blood cells. There are important endothelium-derived vasodilators such as bradykinin, prostacyclin, nitric oxide and, endothelium-derived hyperpolarizing factor. Among these, nitric oxide inhibits cellular growth and migration. Prostacyclin and nitric oxide both exerts potent antiatherogenic and thromboresistant properties by preventing platelet aggregation and cell adhesion. Endothelial vasoconstrictors, such as angiotensin II and endothelin-1, both of which exert prothrombotic and growth-promoting properties counterbalances these effects [17,18].

Ip et al. [19] classified the coronary endothelial damage into three groups and reported that type 3 damage may cause restenosis of the coronary artery. Similarly, Okazaki et al. [2] have classified the endothelial damage in 5 stages and stated that, particularly, type 3 or stage 5 damages had resulted in the development of platelet aggregation and thrombus formation. Factors, such as PDGF, initiate smooth muscle proliferation and migration and as a result, these factors could lead to an early and a late period restenosis [18].

Previous studies have shown that Poloxamer 407 can be utilized to occlude blood vessels during OPCAB [11,15,20,21]. Bouchot et al. [14] have reported that, Poloxamer 407 was effective as a temporary vascular occluder during OPCAB anastomoses and the endothelium of the coronary arteries revealed no functional or a structural damage and the myocardium exhibited no ischemic injury attributable to the polymer plug. In a prospective multicenter randomized clinical study, Wimmer-Greinecker et al. [21] evaluated that Poloxamer 407 could be used safely and effectively in the OPCAB surgery. Poloxamer 407 provided a dry surgical anastomotic field and surgical comfort more frequently than the conventional vessel loop. In addition, anastomotic times were shorter with Poloxamer 407 and major cardiac events were similar in the Poloxamer 407 and vessel loop.

Gertz et al. [6] studied the endothelial cell damage caused by temporary arterial occlusion with surgical clips using scanning and transmission electron microscopy. They have occluded the right carotid artery for 5, 15 and 30 minutes with a surgical clip. They reported that the endothelium of the compressed segments revealed craters and balloons, endothelial cell flattening, discontinuity and desquamation exposing the sub endothelial tissue after 15 or 30 minutes of occlusion. In this study, there was not any crater or balloons, but endothelial cell flattening, discontinuity and desquamation were observed especially in the MV clamp group samples. The duration of occlusion was not more than 10 minutes in our study since we prefered the optimal time for anastomosis during OPCAB.

**Figure 1:**
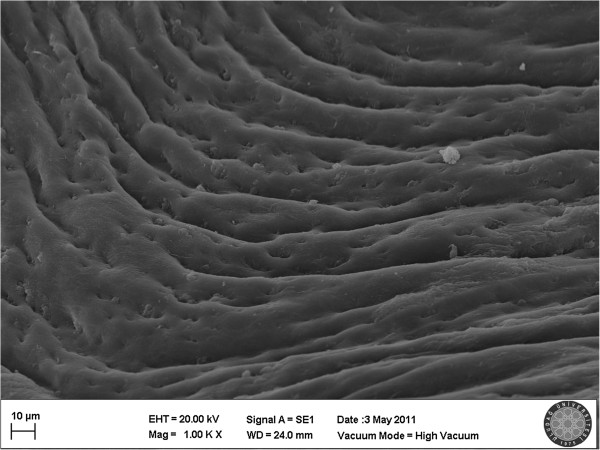
**Scanning electron micrograph of the P 407 group.** The endothelial cells were intact. Blood cells and fibrin were observed adhered to the endothelial surface. The nuclei of the endothelial cells were prominent.

**Figure 2:**
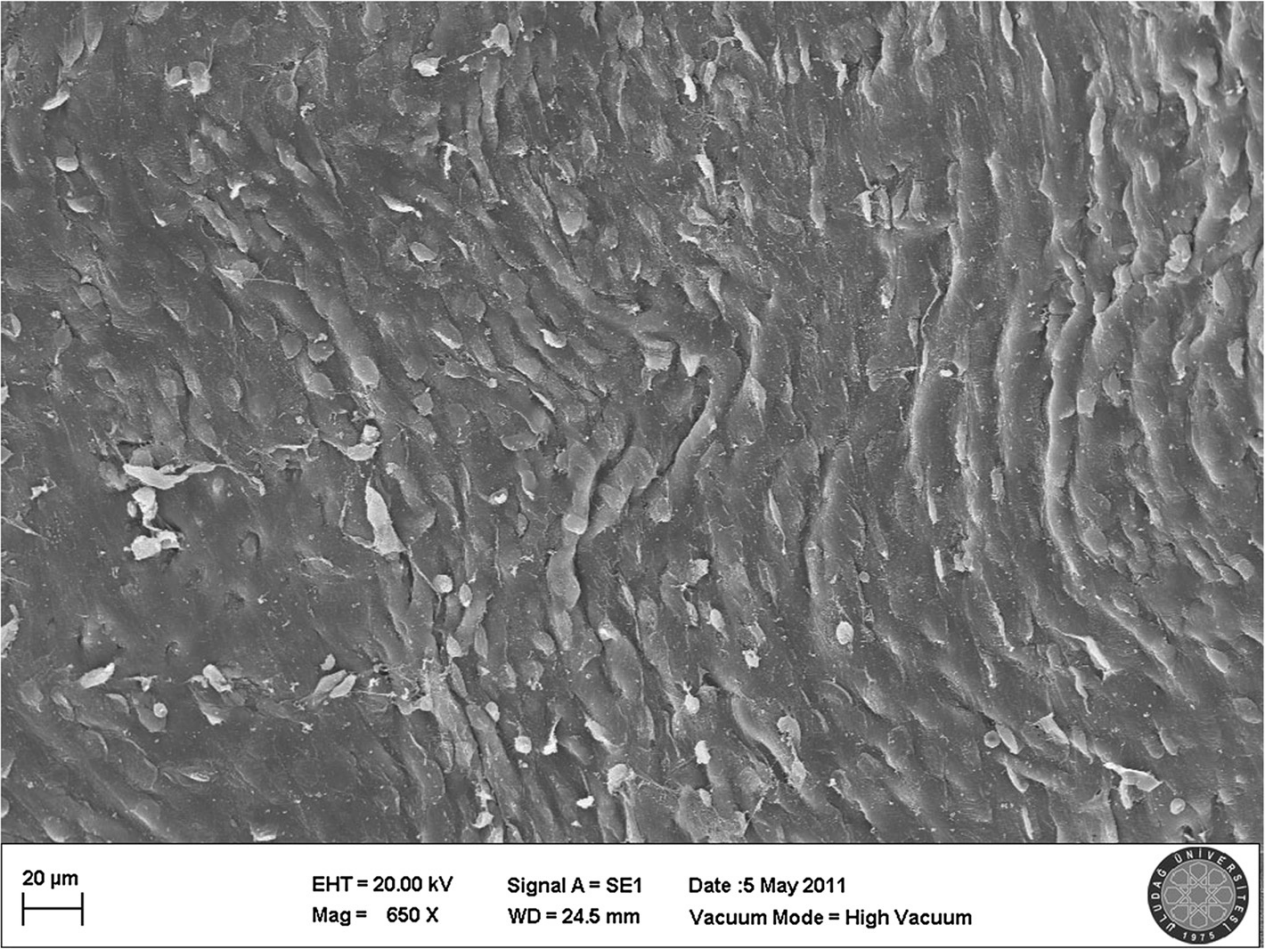
**Scanning electron micrograph of the P 407 group.** In 4 samples, endothelial cells were in contact which each other, but the flattening endothelial cells and decreased in diameter was detected. Other blood cells observed in adhesion to endothelium, damage was evaluated as type 1.

**Figure 3:**
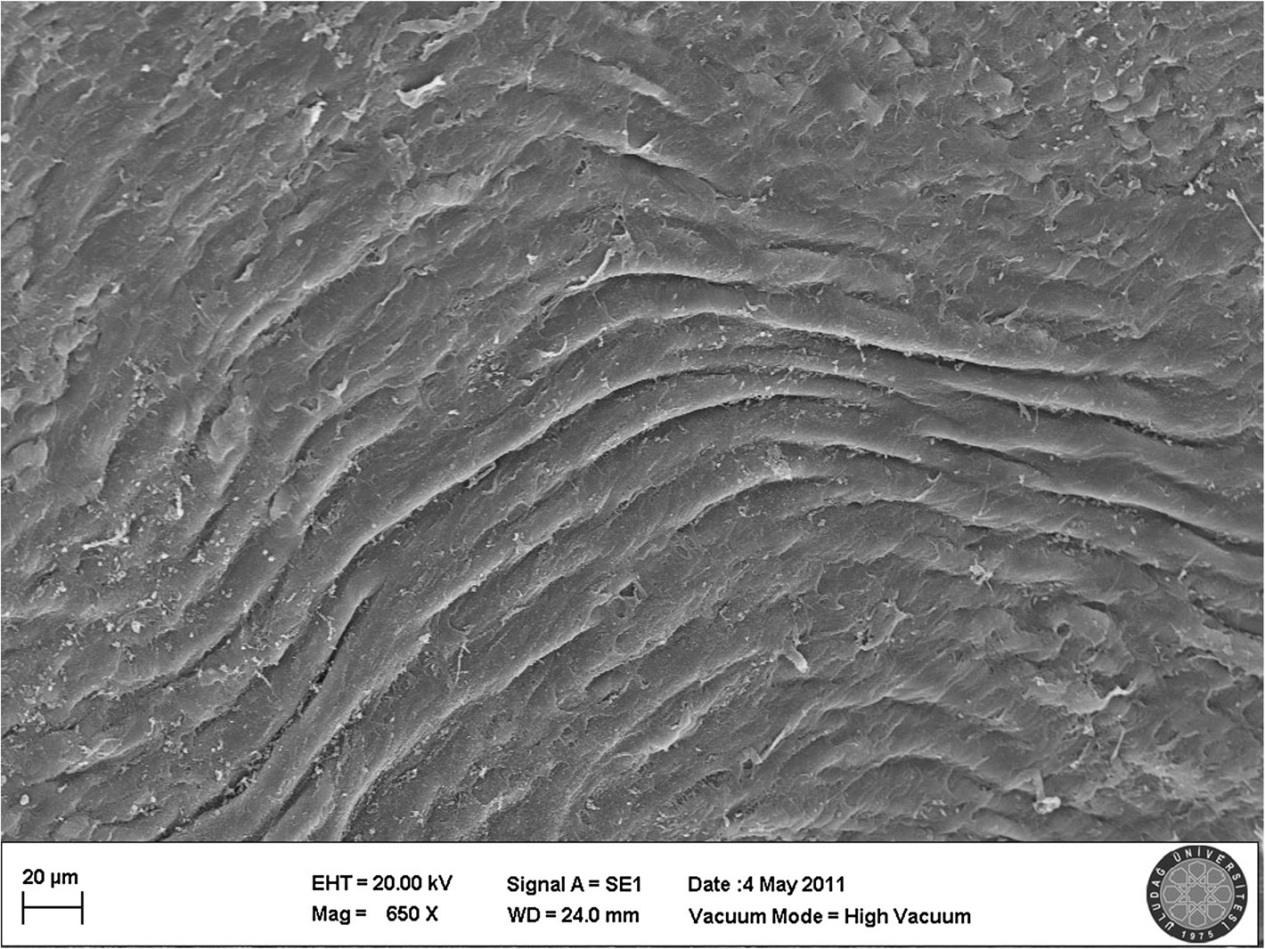
**Scanning electron micrograph of the P 407 group.** In one sample, minimal detachment between the endothelial cells was observed. Damage was evaluated as type 2.

**Figure 4:**
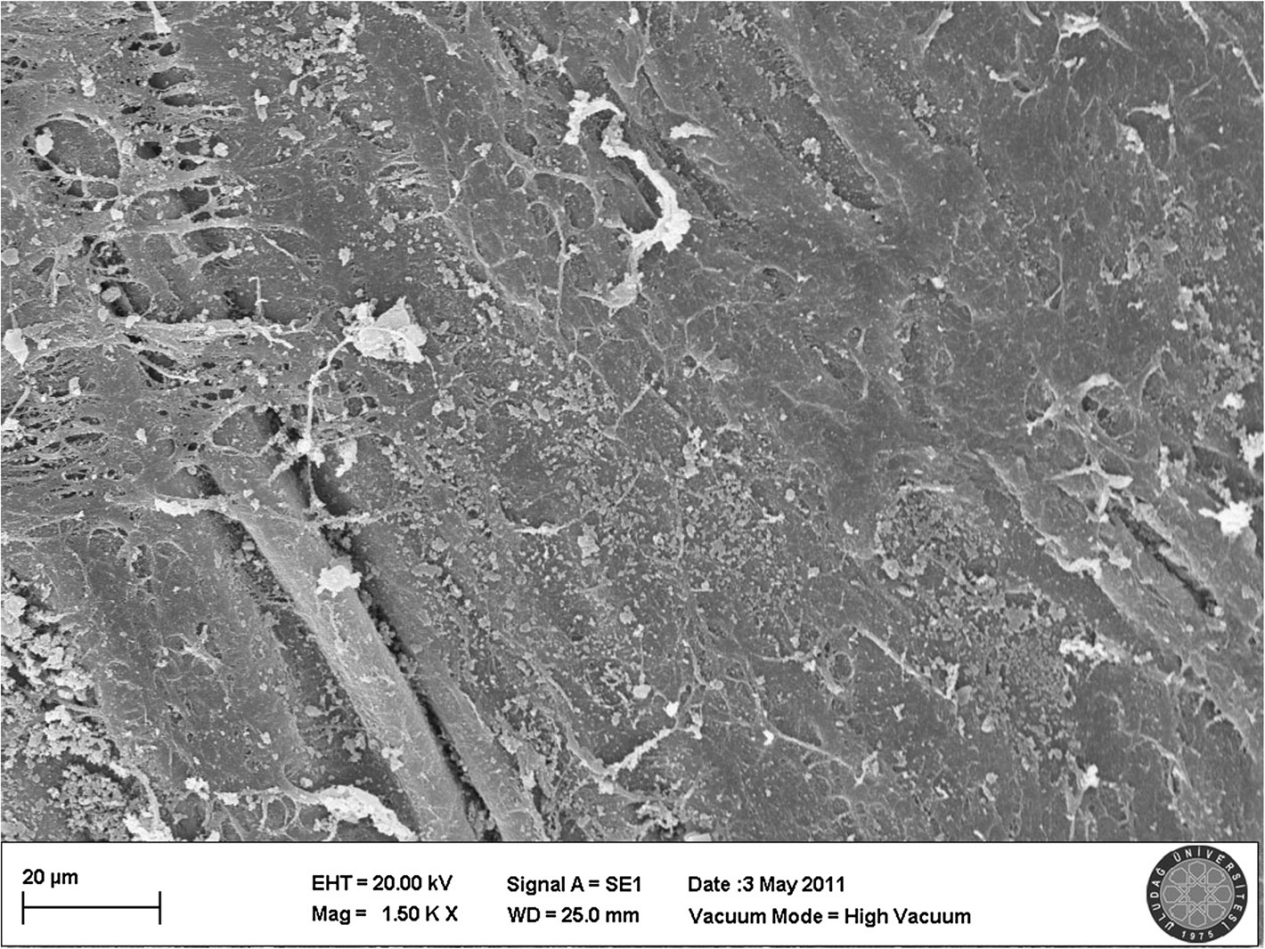
**Scanning electron micrograph of the MV clamp group.** In this sample, detachment of the endothelial cells and patchy areas of peeling subendothelial tissue were observed. Damage was evaluated type 3.

According to the literature, in type 1 damage, integrity of the endothelial cells is reversible and complete recovery is expected in this process [22,23]. In our study, no endothelial damage was observed in 66.7% of the samples of P 407 group (Figure 1). Type 1 was observed in 26.6% (Figure 2), and Type 2 was observed in 6,6% of all samples (Figure 3). These findings are remarkable to determine that the potential damage caused by Poloxamer 407 gel may not give rise to restenosis because in our experiment, 93.3% of the vessels occluded by Poloxamer 407 gel, so the integrity of the endothelium was preserved. Meanwhile, in the MV clamp group type 3 damage was detected in 6.6% of the samples (Figure 4). A statistically significant difference was found between the three groups, and this difference was because of the MV clamp group because no difference was found between P 407 and Control group. On the other hand, the differences between MV clamp–Control group and MV clamp–P 407 groups were statistically significant. These results indicate that MV clamp application has a potential to create endothelial damage. Consequently, it can be suggested that, the vascular occlusion with Poloxamer 407 gel cause no or a minimal damage on the endothelium.

## Conclusions

Deployment of Poloxamer 407 gel was easy, as well as the formation of an occlusive plug was instantaneous. Similarly, dissolution of the Poloxamer 407 plug after the completion of the anastomosis was easy, reliable and quick. There was no report to indicate any embolism to myocardium. Recent clinical studies performed on human beings confirmed the results of previous preclinical experiments indicating that Poloxamer 407 gel is a safe and effective temporary occluder in the OPCAB surgery.

As a result, the finding of the present study clearly demonstrates the safety of Poloxamer 407 gel occlusion and shows lower damage to the endothelium when analyzed at the scanning electron microscopical level.

## Competing interests

The authors declare that they have no competing interests.

## Authors’ contributions

Conception and designed: AG, CE, IC. Analysis and interpretation: AG, CE, FT, TG. Data Collection: AG, CE. Writing the article: AG, CE. Critical revision of the article: AO. Final approval of the article: AG, CE, FT, TG, AO. Optioned funding: not applicable. Overall responsibility: AG. All authors read and approved the final manuscript.
