# Correction: Effects of temporary vascular occluder poloxamer 407 Gel on the endothelium

**DOI:** 10.1186/1749-8090-9-18

**Published:** 2014-01-21

**Authors:** Arif Gücü, Ilkin Cavusoglu, Onder Bozkurt, Cuneyt Eris, Faruk Toktas, Tugrul Goncu, Ahmet Ozyazicioglu

**Affiliations:** 1 Department of Cardiovascular Surgery, Bursa Yuksek Ihtisas Training and Research Hospital, Bursa, Turkey; 2 Department of Histology and Embryology, Uludag University Faculty of Medicine, Bursa, Turkey; 3 Department of Cardiovascular Surgery, Necip Fazil City State Hospital, Kahramanmaras, Turkey

## Correction

After publication of the article by Gücü et al. [1], it was found that Dr Onder Bozkurt had been inadvertently omitted from the author list. The author list has therefore been amended accordingly. The authors apologise for any inconvenience this may cause.
